# APP Deficiency Ameliorates FAD Presenilin 1 F105C and A246E Mutations-induced Mitochondrial Dysfunction in Human Cortical Neurons

**DOI:** 10.7150/ijbs.120062

**Published:** 2026-02-18

**Authors:** Yu-Hsin Yen, Fang Yuan, Daijiao Tang, Jing-Fang Luo, Chen Ming, Phil-Jun Kang, Huanxing Su, Cheong-Meng Chong, Su-Chun Zhang

**Affiliations:** 1 Program in Neuroscience & Behavioral Disorders, Duke-NUS Medical School, 169857 Singapore, Singapore.; 2 GK Goh Centre for Neuroscience, Duke-NUS Medical School, 169857 Singapore, Singapore.; 3 State Key Laboratory of Mechanism and Quality of Chinese Medicine, Institute of Chinese Medical Sciences, University of Macau, Macao SAR 999078, China.; 4 Faculty of Health Sciences, University of Macau, Macao SAR 999078, China.; 5 Ministry of Education Frontiers Science Center for Precision Oncology, University of Macau, Macau SAR 999078, China.; 6 Centre for Cognitive and Brain Sciences, University of Macau, Macao SAR 999078, China.; 7 Center for Neurologic Diseases, Sanford Burnham Prebys Medical Discovery Institute, La Jolla, CA 92037, USA.

**Keywords:** Alzheimer's disease, mitochondrial dysfunction, iPSCs, presenilin 1, CRISPR, amyloid precursor protein

## Abstract

**Background:**

Mitochondrial dysfunction is widely regarded as a central and early feature of Alzheimer's disease (AD) pathology. Prior studies suggest that the accumulation of amyloid precursor protein (APP) within mitochondria contributes to this dysfunction. Mutations in presenilin-1 (PS1), which account for most cases of early-onset familial AD (FAD), have also been shown to impair mitochondrial function. In this study, we investigated how APP influences PS1 mutation-induced mitochondrial dysfunction in human cortical neurons derived from patient induced pluripotent stem cells (iPSCs).

**Methods:**

We analyzed transcriptomic and proteomic datasets from postmortem sporadic AD cortex to identify key dysregulated pathways. To functionally interrogate selected mechanisms, we established a panel of CRISPR/Cas9-engineered human iPSC lines, including PS1 mutant lines (PS1^+/F105C^ and PS1^+/A246E^), an APP knockout derivative (APP^-/-^_PS1^+/F105C^), and their isogenic wild-type controls. These iPSCs were differentiated into cortical neurons for functional studies. Following directed differentiation into cortical neurons, biochemical analyses and super-resolution imaging were conducted to evaluate mitochondrial and neuronal phenotypes.

**Results:**

Analyses of sporadic AD cortical transcriptomes and proteomes identified mitochondrial dysfunction as a prominently altered pathway. In agreement, cortical neurons differentiated from FAD PS1 mutant (F105C and A246E) iPSCs displayed mitochondrial defects and AD-related phenotypes, both of which were mitigated by APP knockout.

**Conclusions:**

These findings provide critical insights into the bridging role of APP in FAD PS1 mutant-mediated mitochondrial dysfunction, advancing our understanding of the cellular mechanisms underlying AD.

## Background

Alzheimer's disease (AD), the most prevalent neurodegenerative disorder, manifests clinically as progressive memory loss and cognitive deterioration 1. As the leading cause of dementia, AD poses a substantial global health burden. Pathologically, AD brains are defined by the presence of extracellular amyloid plaques and intracellular neurofibrillary tau tangles. There are two forms of AD: sporadic AD and familial AD (FAD). Pathogenic variants in presenilin-1 (PS1; *PSEN1*), the catalytic core of γ-secretase, represent the most frequent genetic cause of FAD 2, 3. Heterozygous PS1 mutations lead to early-onset cognitive impairment and recapitulate key clinical and neuropathological features of AD 4, 5. Therefore, cellular models carrying FAD-associated PS1 mutations offer an important platform for investigating the molecular basis of AD.

Among the many proposed mechanisms underlying AD pathogenesis is the mitochondrial hypothesis, which suggests that deficits in mitochondrial energy metabolism and increased oxidative stress drive neuronal dysfunction and degeneration 6, 7. This concept emerged from clinical and neuropathological evidence and has been further reinforced by studies in AD cell and animal models 8-13. Previous studies have reported the localization of amyloid precursor protein (APP), APP fragments, and amyloid-β (Aβ) in the mitochondria, suggesting their potential role in regulating mitochondria function 14-17. However, whether APP mediates the mitochondrial defects caused by FAD PS1 mutations in human cortical neurons—and the mechanisms involved—has not been defined.

Cortical neuron is the primary cell type affected by AD, leading to the characteristic cognitive decline 18. However, analyses of mitochondrial defects in human neurons have been limited. Most AD studies rely on mouse cortical neurons, but species-specific genetic and physiological differences restrict their translational relevance. These limitations arise largely because human cortical neurons are extremely difficult to obtain in adequate quantity and quality for mechanistic studies. To overcome cross-species discrepancies, recent advances in cellular reprogramming now enable the generation of patient-specific induced pluripotent stem cells (iPSCs), which can be differentiated into cortical neurons 19, 20. This approach provides a more physiologically relevant and versatile platform for investigating human cell-type-specific mechanisms of AD *in vitro*.

In this study, we first identified transcriptomic and proteomic alterations associated with mitochondrial dysfunction in cortical tissue from sporadic AD patients. To explore these mechanisms in a controlled human model, we generated cortical neurons from iPSCs carrying two FAD-linked PS1 mutations: F105C, introduced via CRISPR/Cas9 knock-in into a healthy iPSC line, and A246E, derived from a patient's skin fibroblasts. Corresponding isogenic wild-type controls were used to minimize confounding effects from genetic background. Using these human neuronal models, we delineated abnormalities in mitochondrial structure and function, as well as additional AD-related phenotypes. Importantly, we investigated the contribution of APP to these defects and found that APP modulates mitochondrial dysfunction in PS1-mutant neurons. Together, our findings suggest a critical role for APP in mediating mitochondrial and neuronal deficits driven by FAD-associated PS1 mutations.

## Material and Methods

### Human data pre-processing

We utilized the normalized bulk RNA-seq data (syn7391833) derived from brain samples of four Brodmann areas, i.e. the frontal pole (BM10), the superior temporal gyrus (BM22), the parahippocampal gyrus (BM36), and the inferior frontal gyrus (BM44), along with normalized tandem mass tag (TMT) based proteomic data (syn24983526) specifically from BM36, obtained from the Mount Sinai Brain Bank (MSBB) study 21. The cohort comprised 238 female and 126 male participants with diverse racial backgrounds, including European ancestry, African American, Latino, Asian, et al. Sample collection and processing were performed as previously described 21. Briefly, fresh-frozen, never-thawed coronal tissue blocks (∼8 mm thick) were cryo-dissected using a cryostat-equipped reciprocating saw. Tissues from all four brain regions were then cryogenically pulverized using a liquid nitrogen-chilled mortar and pestle. The resulting homogenates were stored at -80°C until nucleic acid and protein extraction.

For RNA sequencing, total RNA was extracted using the RNeasy Lipid Tissue Mini Kit (Qiagen, cat#74804). Library preparation was conducted with the TruSeq RNA Sample Preparation Kit v2 following ribosomal RNA depletion. The libraries were amplified, size-selected, and quality-assessed before being sequenced on an Illumina HiSeq 2500 system to generate 100-nucleotide single-end reads. After quality control and filtering, read counts were normalized using the trimmed mean of M-values (TMM) method and adjusted for covariates, including post-mortem interval (PMI), race, batch, sex, RNA integrity number (RIN), and exonic rate.

For the proteomic analysis, as previously described 22, proteins were extracted from BN36 regions of human brains through tissue lysis, and protein concentration was quantified using BCA assay and visualized on Coomassie-stained short SDS gels. To minimize biological variability, a pooling strategy was adopted using well-characterized specimens with short postmortem intervals that had passed stringent quality control. Proteins were digested sequentially with Lys-C and trypsin, labeled with tandem mass tags (TMT), and fractionated. Subsequent separation was performed via acidic pH reverse-phase liquid chromatography coupled with tandem mass spectrometry (LC-MS/MS). The resulting normalized protein abundances were log2-transformed, and covariates including age and sex were regressed out using the limma package 23. The included participants were characterized using several neuropathological and cognitive assessments, including clinical dementia rating scale (CDR) for cognitive status and dementia severity, Braak staging for neurofibrillary tangle pathology, the Consortium to Establish a Registry for Alzheimer's Disease (CERAD) score for AD neuropathologic changes, and mean plaque density (PlaqueMean), as described in a previous study 24.

### Human data analysis

The samples were further divided into different groups representing multiple disease severity stages based on the four AD traits (Supp Table). For CDR, samples were classified as normal group (CDR = 0), MCI (mild cognitive impairment) group (CDR = 0.5), and AD group (CDR≥ 1). For CERAD, samples were classified according to two kinds of classification. The first one included two groups, the normal group (CERAD =1), and the AD group (CERAD > 1). The second one included four groups: normal group (CERAD =1), definite AD (CERAD = 2), probable AD (CERAD = 3), and possible AD (CERAD = 4). For the Braak score, samples were divided into normal (Braak ≤ 2) and AD (Braak > 2) groups. For PlaqueMean, samples were classified into four groups: normal (PlaqueMean = 0), mild (0 < PlaqueMean ≤ 6), medium (6 < PlaqueMean ≤ 12), and severe (PlaqueMean > 12) groups. For comparisons of gene or protein expression levels across multiple groups, one-way ANOVA followed by Dunnett's test was applied for pairwise comparisons against the normal group. Comparisons between two groups were performed using Student's t-test. Differentially expressed genes (DEGs) and proteins (DEPs) were identified using the limma package 23. Significantly DEGs were defined with an absolute fold change (|FC|) > 1.2 and adjusted p-value (*P_adj_*) < 0.05, based on the Benjamini-Hochberg (BH) method 25. For proteins, a significance threshold of |FC| > 1 and *P_adj_* < 0.05 was adopted. Mitochondria-related functional enrichment analysis for Gene Ontology (GO) terms and pathways was conducted based on the MitoCarta3.0 database 26 using hypergeometric tests.

### Cell culture

The HEK293T cell lines were cultured in DMEM supplemented with 10% FBS and 1% penicillin/streptomycin (Life Technologies) in a humidified atmosphere of 5% CO_2_ at 37 °C. Cells were passaged every 3 days to maintain growth.

### Human iPSC culture

The PS1 F105C iPSC line and its isogenic wild-type control iPSC line (UC-H2-iPSC, Ctrl1) were provided by Professor Huanxing Su at the University of Macau. The PS1 A246E iPSC line (Catalog No. AG25367*D) was acquired from the Coriell Institute. These iPSC lines were cultured in mTeSR1 plus media (STEMCELL Technologies, Cambridge, MA, USA) on Matrigel (Corning, Manassas, VA)-coated plates without feeders. Routine passaging was performed at a 1:10 ratio using Versene (Thermo Fisher Scientific).

### Generation of CRISPR/Cas9 edited iPSC cell line

Isogenic wild-type control (Ctrl2) to PS1 A246E iPSCs was generated via CRISPR/Cas9 guided correction. Briefly, PS1 A246E iPSCs were dissociated into single cells using Accutase. Single strand DNA (ssDNA), guide RNA (gRNA), and TrueCut Cas9 protein (Invitrogen) were transfected into PS1 A246E iPSCs by electroporation using Neon™ Transfection System (Invitrogen). After transfection, the cells were seed in Matrigel-coated 6-well plates at a density of 200 cells/well to ensure that the cell clone was derived from a single cell and grown in mTeSR1 plus media with CloneR™ for eight days. After the single cell colony selection and expansion, the genomic DNA (gDNA) of iPSCs was isolated using Lucigen QuickExtract™ DNA Extraction Solution (Mandel Scientific) and amplified by PCR. Sanger sequencing was used to confirm the corrected site and correction efficiency. The ssDNA, gRNA, and primers are shown in the Supp Table.

PS1 F105C_APPKO iPSCs was generated via CRISPR/Cas9-mediated APP knockout. Briefly, PS1 F105C iPSCs were dissociated into single cells using Accutase. gRNA and TrueCut Cas9 protein (Invitrogen) were transfected into iPSCs by electroporation using Neon™ Transfection System (Invitrogen). After transfection, the cells were seeded in Matrigel-coated 6-well plates at a density of 200 cells/well in mTeSR1 plus media with CloneR™ for eight days. After the single cell colony selection and expansion, the gDNA of iPSCs was isolated using Lucigen QuickExtract™ DNA Extraction Solution (Mandel Scientific) and amplified by PCR. Sanger sequencing was used to confirm the edited site. The gRNA and primers are shown in the Supp Table.

### iPSCs authenticity tests

The gDNA of iPSCs was isolated using Lucigen QuickExtract™ DNA Extraction Solution (Mandel Scientific) for off-target detection. Each off-targets of gRNA was searched using the COSMID tool 19 (http://crispr.bme.gatech.edu). All the off-target sites were confirmed by PCR and sanger sequencing. The primers and off-target analytical results are shown in the Supp Table. Karyotype tests were performed by the Cytogenetics Lab at Singapore General Hospital. Pluripotency of the iPSCs were confirmed by OCT4 and NANOG staining.

### Generation of human cortical neurons and neurospheres from iPSCs

Human cortical neurons were differentiated from iPSCs using dual-SMAD inhibition approach 27. In brief, iPSCs were gently dissociated using Versene into small clusters (avoiding single-cell passaging to maintain survival and differentiation capacity) and cultured on Matrigel-coated 6-well plates until reaching ~10% confluence. On day 0, the medium was changed to Neural Differentiation Medium (NDM) that consists of an equal mix of DMEM/F12 and Neurobasal medium (Gibco), supplemented with N2, B27, and L-glutamax (Gibco), enhanced with 2 μM SB431542 (STEMCELL Technologies) and 2 μM DMH-1 (Tocris). This medium was refreshed every other day until day 7. On the seventh day, cells were transferred at a 1:6 ratio onto a new Matrigel-coated plate and continued in the same medium until day 13, with changes every other day to facilitate the progression to neural progenitors (NPs). From day 14, NPs were suspended in NDM to form neurospheres. On day 20, neurospheres were further dissociated into single cells, seeded on poly-ornithine treated and laminin coated plates or glass coverslips, and cultured in NDM supplemented with 0.1 μM compound E (Tocris) for 5 days to induce neuronal differentiation and further cultured for 25 days in NDM prior to analysis.

### Immunofluorescence staining

Cells were fixed for 15 min with 4% paraformaldehyde (PFA). After three washes with DPBS, cells were blocked in with 5% donkey serum in 0.3% Triton™ X-100 for 1 h and then incubated with primary antibodies overnight at 4°C. After washing three times with DPBS, the samples were incubated with DAPI and fluorophore-conjugated secondary antibodies for 1 h at room temperature. After three washes with DPBS, the image was captured using Nikon Eclipse Ti2 inverted microscope.

### Super-resolution imaging

Super-resolution imaging was performed using a Nikon Eclipse Ti2 inverted microscope, a back-illuminated sCMOS camera (Prime 95B; Photometrics), and the super-resolution system. Super-resolution system consists of Yokogawa CSU-W1 spinning disk confocal, and Live-SR super resolution module from GATACA Systems. The super-resolution method can double the resolution and the optical sectioning capability of confocal microscopy simultaneously 28, 29. All image capture and processing were controlled by MetaMorph software (Molecular Device).

### Western blotting

Cells in the culture plates were rinsed once with ice-cold DPBS and lysed in the RIPA buffer containing 1% PMSF and 1% protease/phosphatase inhibitor cocktail (Thermo Fisher Scientific, Waltham, MA, USA) for 30 min at 4 °C, followed by centrifugation at 12,500 g for 20 min at 4 °C. Lysates in a 1× sample buffer were boiled for 5 min at 95 °C for denaturation and separated by sodium dodecyl sulfate-polyacrylamide gel electrophoresis. The target proteins were detected by western blotting with their respective specific antibodies, and α-Tubulin was used as an internal control. The blot was visualized using an ECL kit (GE Healthcare), according to the manufacturer's instructions. The intensity of the bands was quantified using Image Lab software.

### TMRE staining

The mitochondrial membrane potential was measured by TMRE staining (Thermo Fisher Scientific). The neurons were incubated with TMRE dye (500 nM in medium) at 37 °C for 20 min. After that, the cells were rinsed twice with medium. The image was captured using Nikon Eclipse Ti2 inverted microscope. The fluorescence intensity was determined by ImageJ. All values were normalized to the control group.

### Human amyloid beta ELISA

The culture medium of iPSC-derived cortical neuron conditioned for 4 days was collected for measurement of Aβ levels. The levels of secreted Aβ 42 and Aβ40 were measured using Human Amyloid beta Quantikine ELISA kits (R&D kits Systems DAB140B, DAB142), according to the manufacturer's instructions.

### Immunoprecipitation

Immunoprecipitation was performed by Pierce™ Classic MagneticIP/Co-IP Kit according to the manufacturer (Thermo Fisher Scientific, Waltham, MA, USA). In brief, cells were lysed in IP Lysis/Wash Buffer. Following centrifugation at 13,000 × g, supernatants were incubated with primary antibody overnight at 4ºC. Antibody mixtures were mixed with Protein A/G magnetic beads (Thermo Fisher Scientific) and incubated for 1 h at room temperature. After washing with IP Lysis/Wash Buffer, Proteins in beads were eluted in Elution Buffer and analyzed using western blotting.

### Seahorse assay

The oxygen consumption rate (OCR) was assessed using the Agilent Seahorse XFe96 Analyzer and the XF Cell Mito Stress Test kit according to the manufacturer's protocol (Agilent Technologies, USA). In brief, day 20 neurospheres were dissociated into single cells and seeded on poly-ornithine-treated and laminin-coated Seahorse plates. After neuronal differentiation, these cells were subjected to the Seahorse assay. Subsequently addition of oligomycin (1 μM), FCCP (0.5 μM), and rotenone/antimycin A (1 μM) was performed to assess key parameters of mitochondrial metabolic function.

### Statistical analysis

Statistical analyses were conducted using GraphPad Prism version 10.2.0 statistical software (GraphPad Software, Inc., San Diego, CA, USA). All experiments were carried out in at least biological triplicates, and the results were reported as mean± standard error of the mean (SEM). Statistical analysis was carried out using a one-way analysis of variance, followed by Tukey's multiple comparison. Statistical analysis between the two groups was performed using the two-tailed paired Student's t test. A p-value of less than 0.05 was deemed significant.

## Results

### Sporadic AD patients exhibit mitochondrial dysfunction in BM36 region (parahippocampal gyrus)

**Figure 1:**
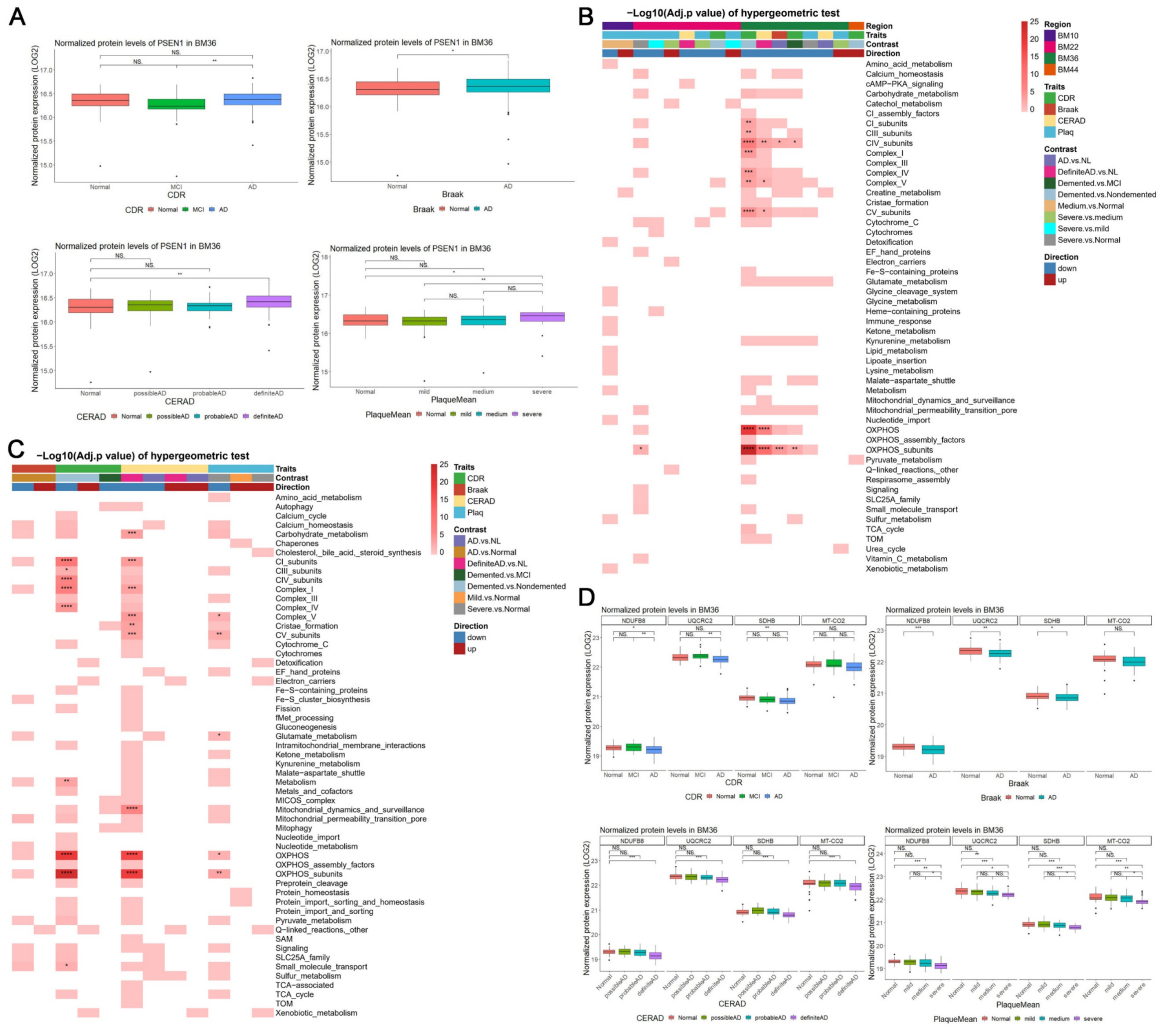
** Transcriptomic and proteomic data showed mitochondrial dysfunction in BM36 of AD patients. (A)** The normalized protein expression levels of *PSEN1* in BM36 across different groups according to four AD traits. **(B)** The significant DEGs [abs(*FC*) > 1.2, *P_adj_* < 0.05] in four brain areas enriched in mitochondrial pathways [-Log10(*P_adj_*) of hypergeometric tests]. **(C)** The significant DEPs [abs(*FC*) > 1, *P_adj_* < 0.05] in BM36 enriched in mitochondrial pathways [-Log10(*P_adj_*) of hypergeometric tests]. **(D)** The normalized protein expression levels of NDUFB8, UQCRC2, SDHB, and MT-CO2 in BM36 across different groups according to four AD traits. The comparisons of gene or protein expression levels across multiple groups were conducted using one-way ANOVA followed by Dunnett's test for pairwise comparisons against the normal group. For comparisons between two groups, Student's t-test was employed. *P < 0.05, **P < 0.01, ***P < 0.001, and ****P < 0.0001 were considered significantly different.

To investigate the potential changes of mitochondrial function in the cerebral cortex during AD, we utilized existing bulk RNA-seq and TMT-based proteomic data of human postmortem brains from the Mount Sinai Brain Bank (MSBB) RNA-seq study 21. The postmortem brain specimens were extracted from four Brodmann areas, i.e., the frontal pole (BM10), the superior temporal gyrus (BM22), the Parahippocampal gyrus (BM36), and the inferior frontal gyrus (BM44). BM36 obtained the greatest number of significant differential expression genes (DEGs) [abs(FC) > 1.2 and *P_adj_* < 0.05], including 3,332 upregulated and 2,504 downregulated DEGs (Supp Figures 1A-B). Meanwhile, we also identified 1,308 upregulated and 654 downregulated differential expression proteins (DEPs) which showed consistent alterations with the corresponding DEGs in BM36 [abs(FC) > 1 and *P_adj_* < 0.05] (Supp Figures 1C-D). Notably, the expression of both mRNA and protein levels of *PSEN1* was significantly upregulated in AD groups in BM36 according to four AD traits (Supp Figure 2A; Figure 1A). We further found that the significant DEGs from four brain areas and the significant DEPs in BM36 were enriched in the mitochondrial pathways included in the MitoCarta3.0 database 26 (Figures 1B-C), especially those involved in mitochondrial complex and oxidative phosphorylation (OXPHOS) (*P_adj_* < 0.05). In the BM36 region, genes encoding components of the mitochondrial respiratory chain, including NDUFB8, UQCRC2, COX2, and SDHB, were downregulated at both the mRNA and protein levels in AD samples (Figure 1D; Supp Figures 2B-E). These results suggest the presence of mitochondrial dysfunction in the cerebral cortex of sporadic AD patients, particularly in the BM36 region, which shows a strong association with elevated PS1 levels.

### FAD PS1 F105C and A246E mutant cortical neurons display hyperphosphorylated tau accumulation and increased Aβ generation

**Figure 2:**
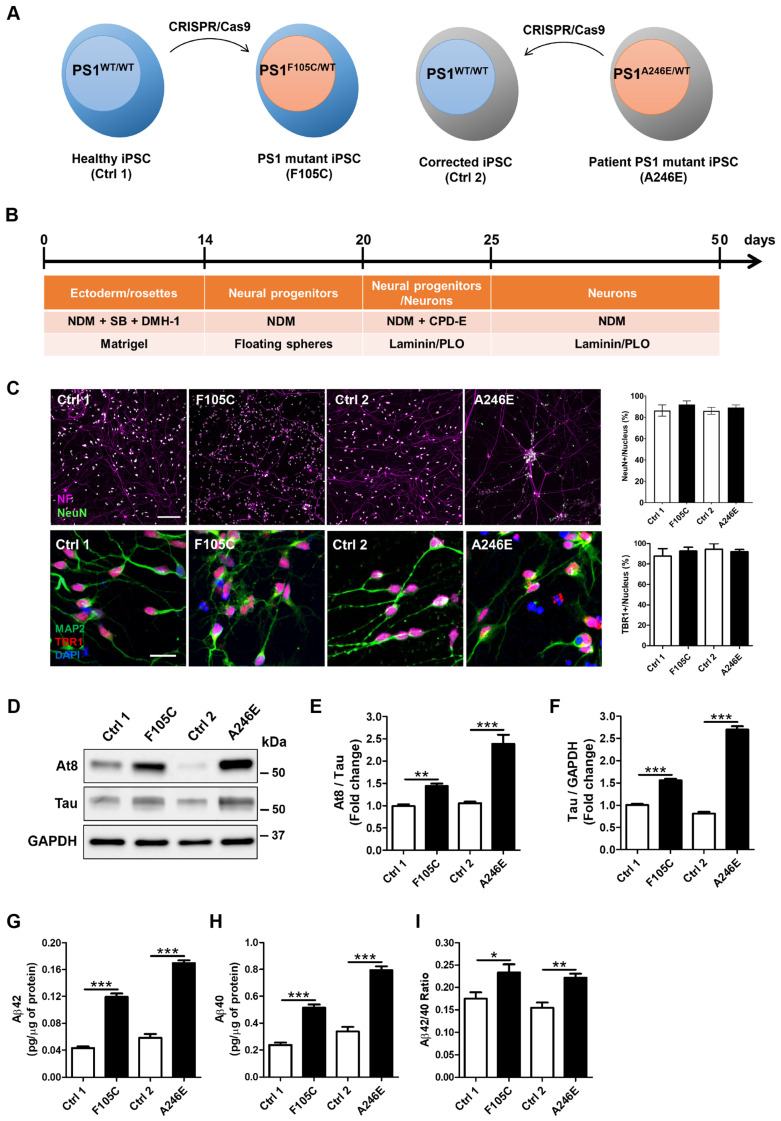
** AD-related features in human FAD PS1 mutant iPSC-derived cortical neurons. (A)** Schematic representation of the generation of two isogenic pairs of iPSC lines (Ctrl1 and F105C; Ctrl2 and A246E) by CRISPR/Cas9 gene editing. **(B)** The workflow for the cortical neuronal differentiation of iPSCs. **(C)** Neurofilament (NF), NeuN, MAP2, and TBR1 immunostaining of iPSC-derived cortical neurons and the percentages of NeuN-positive and TBR1-positive neurons (30 neurons per group, n = 3). Scale bar = 100 μm and 25 μm. **(D)** Representative western blotting of At8, Tau, and GAPDH in iPSC-derived neurons. **(E)** The changes in AT8/Tau and Tau/GAPDH **(F)** were quantified by western blotting analysis (n = 3). Data are represented as mean±SEM. **P < 0.01 and ***P < 0.005 were considered significantly different. **(G) (H)** ELISA quantification of Aβ42 and Aβ40 secreted from iPSC-derived neurons (n = 5). Data are represented as mean±SEM. *P < 0.05, **P < 0.01, and ***P < 0.005 were considered significantly different. **(I)** Mutation dependent changes in Aβ42/40 ratio in these neurons. Data are represented as mean±SEM. *P < 0.05, **P < 0.01, and ***P < 0.005 were considered significantly different.

Given the pathogenic role of PS1 mutations in AD development, we used CRISPR/Cas9 to generate two isogenic pairs of human iPSC lines (see Figure 2A, Supp Figure 3A). The first pair consists of a healthy donor iPSC line (Ctrl1) and a CRISPR-engineered knock-in line carrying the PS1F105C mutation, caused by a T→G substitution at nucleotide 314 that results in a phenylalanine-to-cysteine change at residue 105 within the loop between PS1 transmembrane domains 1 and 2. The second pair comprises iPSCs from a patient with a PS1 A246E mutation and its CRISPR-corrected isogenic wild-type counterpart; this mutation arises from a C→A substitution at nucleotide 737, leading to an alanine-to-glutamic acid change at residue 246 in transmembrane domain 6 of PS1 (Supp Figure 3B). These paired mutant-wild-type lines enable direct comparison of the cellular effects of distinct PS1 mutations within controlled genetic backgrounds.

Using CRISPR/Cas9 to generate isogenic cell lines removes background genetic variation and allows us to directly compare the effects of the PS1 mutations. DNA sequencing confirmed that the F105C mutation was successfully introduced into the Ctrl1 line, and the A246E mutation in the patient line was corrected to create its wild-type isogenic control (Ctrl2) (Supp Figure 3C). After 10 passages, all iPSC lines showed typical morphological characteristics of pluripotent stem cells as well as high expression of pluripotency markers Oct4 and Nanog (Supp Figure 3D). All lines retained normal karyotypes (Supp Figure 3E). These isogenic pairs of iPSCs were used throughout the experiments.

After 25 days of neuronal differentiation (Figure 2B), immunostaining confirmed that both mutant and isogenic iPSC-derived lines successfully differentiated into TBR1-positive cortical neurons (Figure 2C). After 50 days of neuronal differentiation, these neurons exhibited co-expression of the neuronal marker MAP2, along with mature neuron markers such as neurofilament and NeuN (Figure 2C). Furthermore, cortical neurons harbouring the PS1 mutation recapitulated key pathological hallmarks of AD. One critical hallmark, tau pathology, characterized by the accumulation of hyperphosphorylated tau, was evident. Western blotting analysis showed a significant increase in both tau protein levels and the AT8/tau ratio in PS1 mutant cortical neurons compared to isogenic WT controls (Figures 2D-F). Additionally, Aβ plaques are extracellular aggregates of Aβ. ELISA assays indicated a significant elevation in the levels of Aβ42 and Aβ40, as well as an increased Aβ42/Aβ40 ratio in the culture medium of PS1 mutant cortical neurons (Figures 2G-I). Our results suggest that cultured PS1 mutant cortical neurons exhibit both tau hyperphosphorylation and an increased propensity to generate Aβ aggregates, similar to observations made in AD patients.

### FAD PS1 F105C and A246E mutant cortical neurons exhibit mitochondrial dysfunction

**Figure 3:**
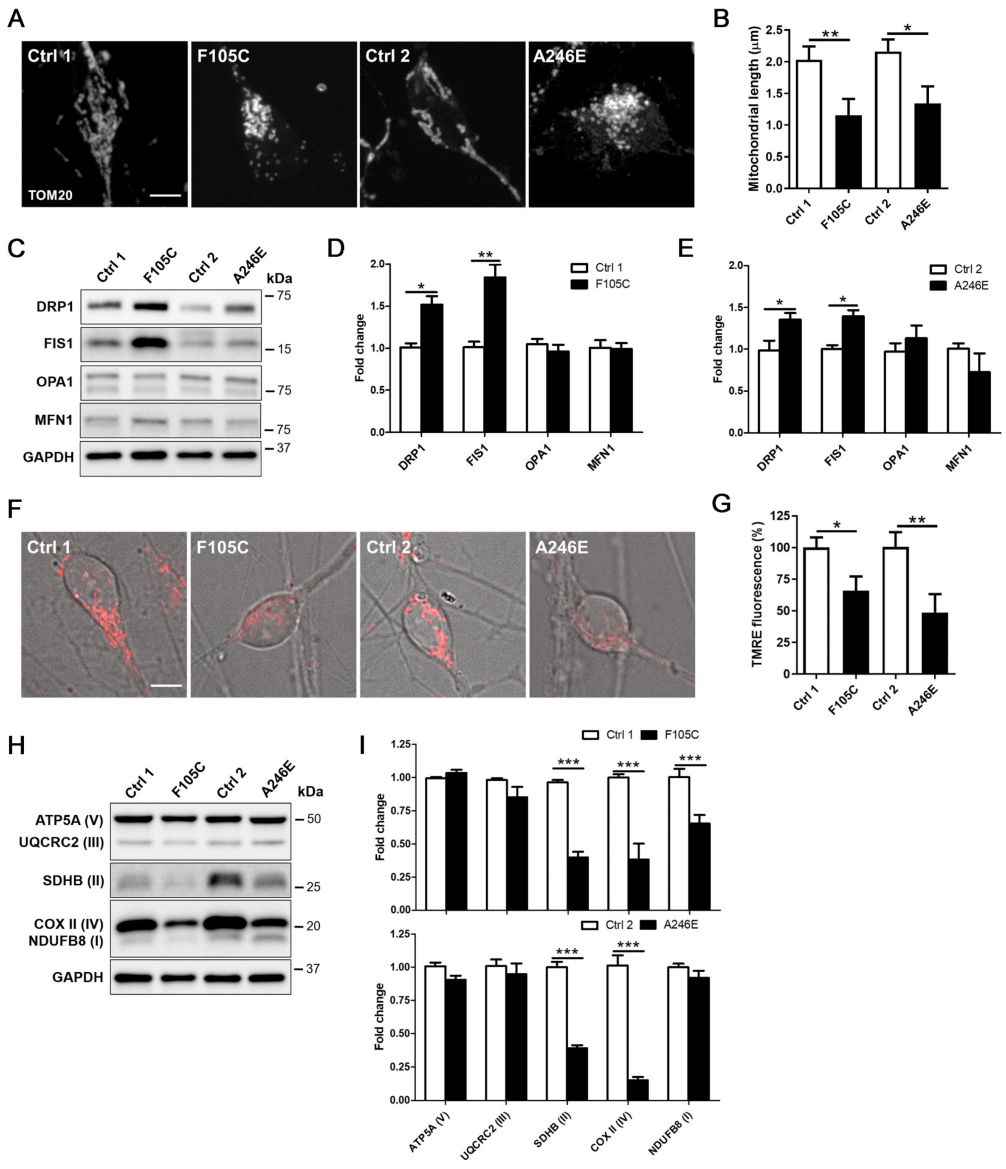
** Mitochondrial dysfunction in human FAD PS1 mutant iPSC-derived cortical neurons. (A)** TOM20 immunostaining of iPSC-derived cortical neurons. Scale bar = 5 μm. **(B)** The quantification of the mitochondrial length (30 neurons per group, n = 3). Data are represented as mean±SEM. *P < 0.05 and **P < 0.01 were considered significantly different. **(C)** Representative western blotting of DRP1, FIS1, OPA1, MFN1, and GAPDH in iPSC-derived neurons. **(D)(E)** The changes in DRP1/GAPDH, FIS1/GAPDH, OPA1/GAPDH, and MFN1/GAPDH were quantified by Western blotting analysis (n = 3). Data are represented as mean±SEM. *P < 0.05 and **P < 0.01 were considered significantly different. **(F)(G)** Mitochondrial membrane potential of iPSC-derived cortical neurons was determined by TMRE staining with 30 neurons quantified per group for each of three independent biological replicates (n = 3). Scale bar = 5 μm. Data are represented as mean±SEM. *P < 0.05 and **P < 0.01 were considered significantly different. **(H)** Representative western blotting of ATP5A (V), UQCRC2 (III), SDHB (II), COXII (IV), NDUFB8 (I), and GAPDH in iPSC-derived neurons. **(I)** The changes in ATP5A (V)/GAPDH, UQCRC2 (III)/GAPDH, SDHB (II)/GAPDH, COXII (IV)/GAPDH, and NDUFB8 (I)/GAPDH were quantified by Western blotting analysis (n = 3). Data are represented as mean±SEM. ***P < 0.005 was considered significantly different.

Mitochondrial dysfunction is a core pathological feature in AD. Through immunofluorescence staining in PS1 mutant and control cortical neurons for mitochondria biomarker Translocase of the outer mitochondrial membrane complex subunit 20 (TOM20), we found that control neurons exhibited long mitochondria throughout soma and neurites. In both PS1 mutant cortical neurons carrying F105C and A246E mutations, we observed fragmented mitochondria in the whole cell body (Figure 3A). Average mitochondrial length in PS1 mutant neurons was significantly shorter than isogenic control neurons (Figure 3B). Due to the morphological changes of mitochondria, western blotting analysis was used to examine changes in the expression of mitochondria fusion and fission proteins. As shown in Figures 3C-E, PS1 mutant cortical neurons exhibited increased levels of fission proteins DRP1 and FIS1, but not fusion proteins OPA1 and MFN1.

The decline of mitochondrial membrane potential is a typical hallmark of mitochondrial dysfunction. Thus, we further examined the mitochondrial state through TMRE (tetramethylrhodamine, ethyl ester) staining assay. As compared with their isogenic WT cortical neurons, both PS1 mutant cortical neurons showed a reduction of mitochondrial membrane potential, revealed through decreased TMRE fluorescence intensity (Figures 3F-G). As compared with isogenic WT controls, PS1 F105C cortical neurons exhibited around 25% decrease in TMRE intensity, whereas PS1 A246E cortical neurons had around 65% decrease in TMRE intensity compared to their respective isogenic WT controls. Further examination of mitochondrial OXPHOS-related proteins showed that the levels of NDUFB8 (I), UQCRC2 (III), and COX II (IV) in PS1 F105C neurons were less than Ctrl1 neurons, whereas PS1 A246E neurons had decreased levels of UQCRC2 (III) and COX II (IV) as compared with Ctrl2 neurons (Figures 3H-I). These results indicate mitochondrial dysfunction in cultured PS1 mutant cortical neurons.

### FAD PS1 F105C and A246E mutant cortical neurons display increased APP in mitochondria

**Figure 4:**
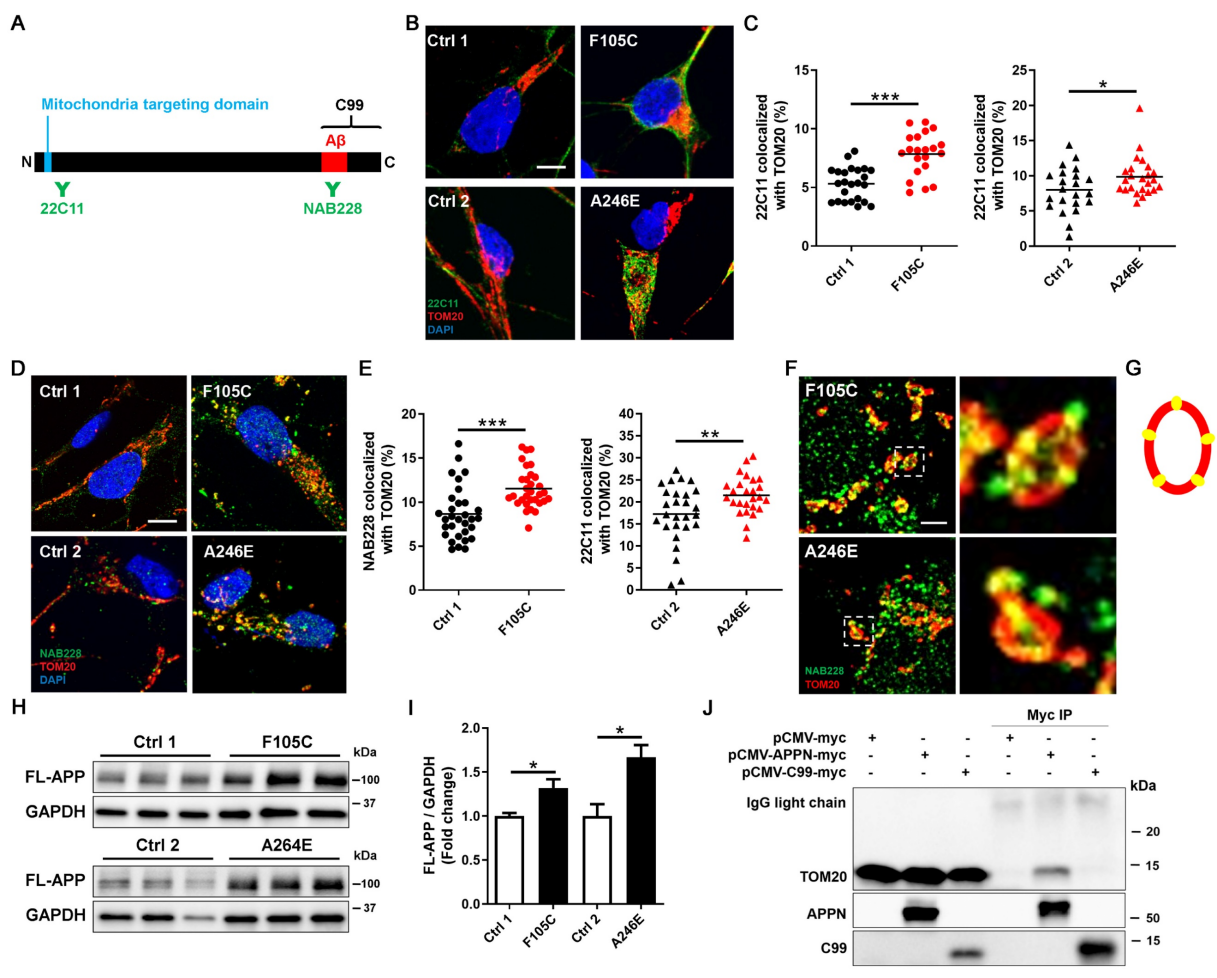
** The increased mitochondrial distribution of APP in human FAD PS1 mutant iPSC-derived cortical neurons. (A)** Schematic representation of the APP protein. **(B)** 22C11 and TOM20 immunostaining of iPSC-derived cortical neurons. Scale bar = 5 μm. **(C)** The quantification of colocalization of 22C11 and TOM20 (more than 25 neurons per group). *P < 0.05 and ***P < 0.005 were considered significantly different. **(D)** NAB228 and TOM20 immunostaining of iPSC-derived cortical neurons. Scale bar = 5 μm. **(E)** The quantification of colocalization of NAB228 and TOM20 (20 neurons per group). **P < 0.01 and ***P < 0.005 were considered significantly different. **(F)** The amplified image of NAB228 and TOM20 immunostaining of iPSC-derived cortical neurons. Scale bar = 1 μm. **(G)** The distribution of NAB228 on mitochondrial outer membrane. **(H)** Representative western blotting of FL-APP and GAPDH in iPSC-derived neurons. **(I)** The changes in FL-APP/GAPDH were quantified by western blotting analysis (n = 3). Data are represented as mean±SEM. *P < 0.05 was considered significantly different. **(J)** The lysates from pCMV-myc, pCMV-APPN-myc, and pCMV-C99-myc transfected HEK293T cells were immunoprecipitated with the anti-myc antibody and obtained IPs were analyzed for the interaction between TOM20 and APP fragments.

It is known that full-length APP (FL-APP) carries a mitochondria-targeting domain near its N-terminus and a γ-secretase cleaved C99 fragment at its C-terminus. To investigate the role of APP in mitochondria defects in PS1 mutant neurons, we examined APP localization using immunofluorescence staining with two antibodies that target the APP N-terminus (22C11) and APP C-terminus (NAB228) (Figure 4A). Super-resolution imaging showed that more APP puncta, marked by 22C11 and NAB228 antibodies, were highly co-localized with mitochondria marker TOM20 in PS1 mutant neurons than in isogenic controls (Figures 4B-E). TOM20 is one of the subunits of translocases in the mitochondrial outer membrane, known to recognize, bind, and import proteins with a mitochondrial targeting sequence 30. Single plane images showed that in PS1 mutant neurons, TOM20 displayed as a hollow oval, and NAB228 was located in the TOM20 oval (Figure 4F-G), suggesting that C-terminus of APP is distributed in the mitochondrial outer membrane. Western blotting showed that the expression of FL-APP was significantly higher in PS1 mutant neurons than isogenic control (Figure 4H and 4I).

In addition to FL-APP, NAB228 also recognizes C99. To investigate whether the C99 has the capacity to bind the mitochondrial outer membrane, we overexpressed myc-tagged N-terminal APP fragment without the C99 segment and myc-tagged C99 in HEK293T cells and performed immunoprecipitation to assess the interaction between these APP fragments and TOM20. The results showed that N-terminal APP fragment containing a mitochondria-targeting domain co-precipitated with TOM20, whereas C99 could not bind to TOM20 (Figure 4J). These data suggest that FL-APP is distributed to the outer mitochondrial membrane in PS1 mutant cortical neurons via its mitochondria-targeting domain.

### APP knockout reduces mitochondrial dysfunction and hyperphosphorylated tau accumulation in FAD PS1 F105C mutant cortical neurons

**Figure 5:**
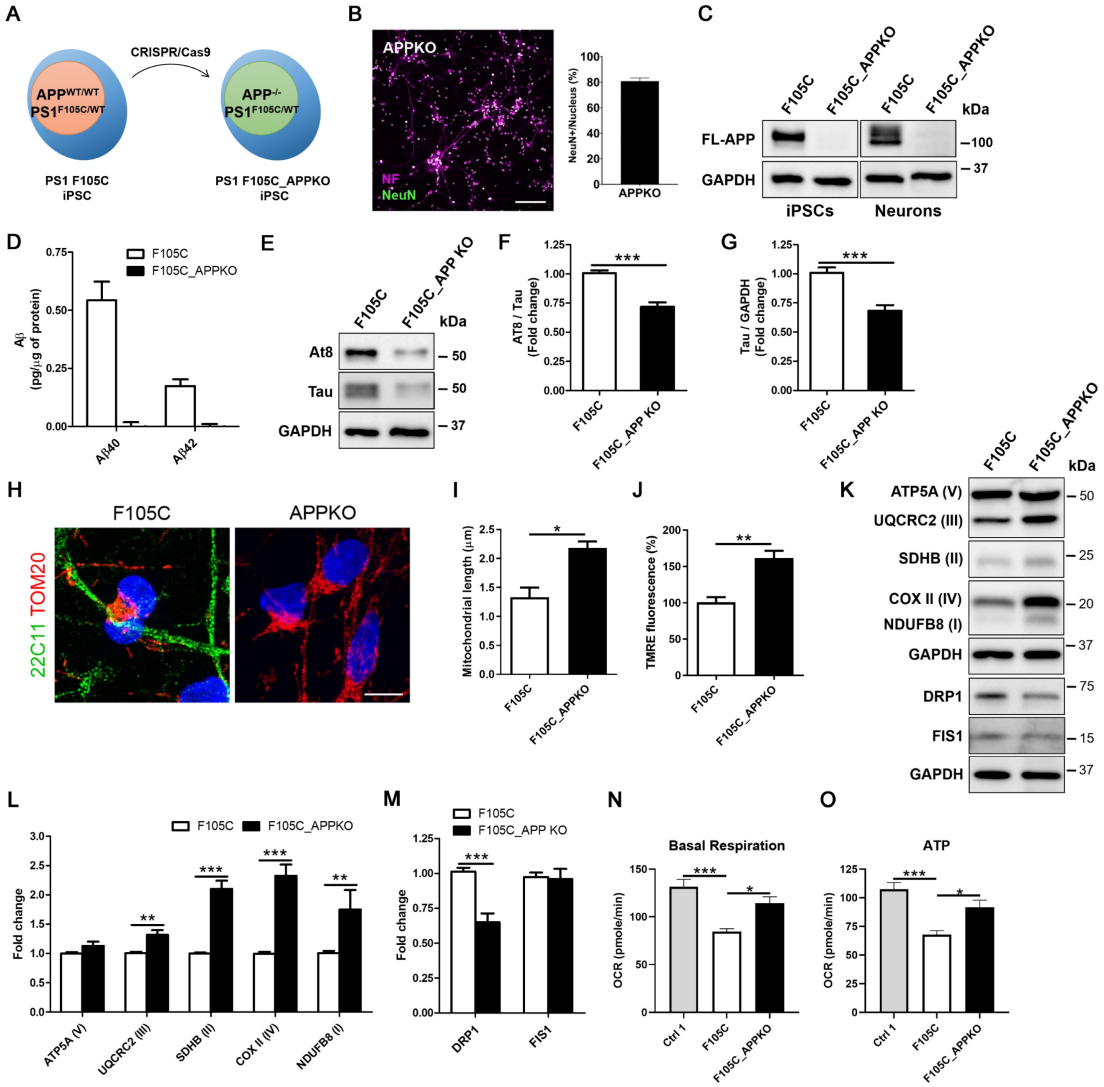
** The effect of APPKO on AD-related characteristics in PS1 mutant iPSC-derived cortical neurons. (A)** Schematic representation of the generation of F10C_APPKO iPSC line by CRISPR/Cas9 gene editing. **(B)** NF and NeuN immunostaining of iPSC-derived cortical neurons and the percentages of NeuN-positive neurons (n = 3). Scale bar = 100 μm. **(C)** Representative western blotting of FL-APP and GAPDH in iPSCs and neurons. **(D)** ELISA quantification of Aβ42 and Aβ40 secreted from iPSC-derived neurons (n = 5). Data are represented as mean±SEM. **(E)** Representative western blotting of At8, Tau, and GAPDH in iPSC-derived neurons. **(F)** The changes in AT8/Tau and **(G)** Tau/GAPDH were quantified by western blotting analysis (n = 3). Data are represented as mean±SEM. ***P < 0.005 was considered significantly different. **(H)** 22C11 and TOM20 immunostaining of iPSC-derived cortical neurons. Scale bar = 10 μm. **(I)** The quantification of the mitochondrial length (30 neurons per group, n = 3). Data are represented as mean±SEM. *P < 0.05 was considered significantly different. **(J)** Mitochondrial membrane potential of iPSC-derived cortical neurons was determined by TMRE staining (30 neurons per group, n = 3). Data are represented as mean±SEM. **P < 0.01 was considered significantly different. **(K)** Representative western blotting of ATP5A (V), UQCRC2 (III), SDHB (II), COXII (IV), NDUFB8 (I), DRP1, FIS1, and GAPDH in iPSC-derived neurons. **(L)** The changes in ATP5A (V)/GAPDH, UQCRC2 (III)/GAPDH, SDHB (II)/GAPDH, COXII (IV)/GAPDH, and NDUFB8 (I)/GAPDH were quantified by western blotting analysis (n = 3). Data are represented as mean±SEM. **P < 0.001 and ***P < 0.005 were considered significantly different. **(M)** The changes in DRP1/GAPDH and FIS1/GAPDH were quantified by western blotting analysis (n = 3). Data are represented as mean±SEM. ***P < 0.005 was considered significantly different. **(N)** Basal respiration and **(O)** ATP were quantified from OCR curve (n = 3). Data are represented as mean±SEM. *P < 0.05 and ***P < 0.005 were considered significantly different.

To investigate whether APP contributes to mitochondria dysfunction in PS1 mutant neurons, we used CRISPR/Cas9 to perform APP knockout (KO) in PS1 F105C iPSCs (Figure 5A). The immunofluorescence staining showed that APP KO did not affect the differentiation and maturation of cortical neurons generated from iPSCs (Figure 5B). Western blotting further confirmed the absence of APP in F105C APPKO iPSCs and neurons as compared to F105C iPSCs and neurons (Figure 5C). In addition, ELISA assay showed that Aβ42 and Aβ40 were not detected in culture medium from F105C APPKO cortical neurons (Figure 5D).

Interestingly, APPKO significantly reduced tau and AT8/tau ratio in PS1 F105C cortical neurons (Figures 5E-F). Through immunofluorescence staining, we found that F105C APPKO cortical neurons displayed long mitochondria in the whole cell body (Figure 5G). Average mitochondrial length in APPKO cortical neurons was significantly longer than isogenic PS1 F105C cortical neurons (Figure 5H). The results of TMRE staining showed that APPKO cortical neurons had higher mitochondria membrane potential than PS1 F105C cortical neurons (Figure 5I). Compared with PS1 F105C cortical neurons, APPKO cortical neurons exhibited an increase in mitochondrial OXPHOS-related proteins including NDUFB8 (I), SDHB (II), UQCR2 (III), and COX II (IV) via western blotting analysis (Figures 5K-L). Additionally, decreased level of fission protein DRP1 also was also found in APPKO cortical neurons (Figures 5K-M). To further validate the effect of APP KO, we analysed the mitochondrial metabolic activity by measuring oxygen consumption rate (OCR) by Seahorse XF technology. As shown in Figures 5N-O, PS1 F105C neurons had lower basal respiration and ATP as compared to Ctrl 1 neurons, whereas APP KO increased in these parameters in PS1 F105C neurons. These results suggest that APP likely mediates the role of mutant PS1 in mitochondrial dysfunction and hyperphosphorylated tau accumulation in cortical neurons.

### APP knockout reduces phosphorylated tau and Aβ42 in FAD PS1 F105C mutant cortical neurospheres

**Figure 6:**
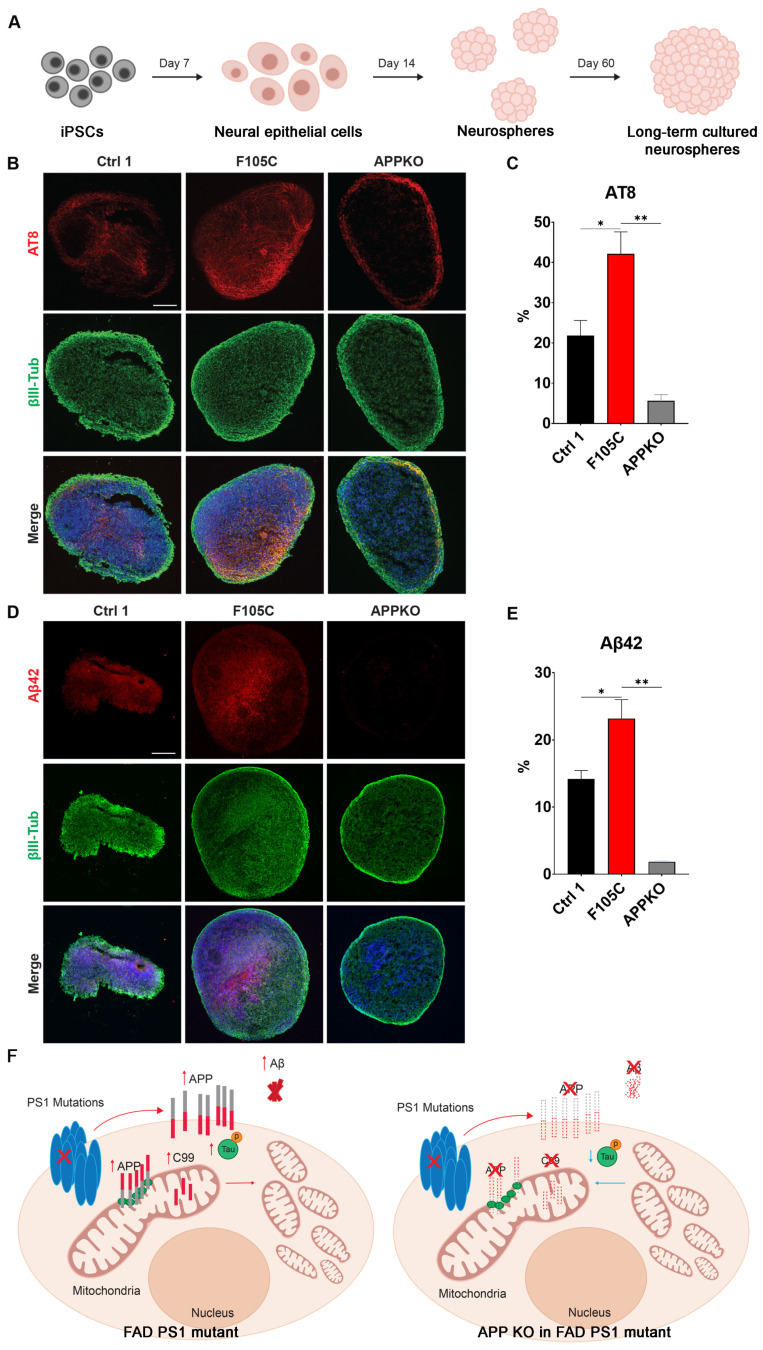
** The effect of APPKO on phosphorylated tau and Aβ42 in PS1 mutant iPSC-derived cortical neurospheres. (A)** The workflow for the cortical neurosphere differentiation of iPSCs. **(B)** AT8 and βIII-Tub immunostaining of iPSC-derived cortical neurospheres. Scale bar = 100 μm. **(C)** The changes in AT8 in neurospheres were quantified by Image J (n = 3). Data are represented as mean±SEM. *P < 0.05 and **P < 0.01 were considered significantly different. **(D)** Aβ42 and βIII-Tub immunostaining of iPSC-derived cortical neurospheres. Scale bar = 100 μm. **(E)** The changes in Aβ42 in neurospheres were quantified by Image J (n = 3). Data are represented as mean±SEM. *P < 0.05 and **P < 0.01 were considered significantly different. **(F)** The proposed mechanism for the mitochondrial functional defects and phosphorylated tau accumulation caused by PS1 mutations and APP.

To evaluate long-term changes, cortical neurospheres derived from isogenic WT control, PS1 F105C, and F105C-APPKO iPSCs were maintained in a three-dimensional culture system for 60 days (Figure 6A). At this stage, neurospheres exhibit features of neuronal maturation while remaining below the size threshold at which extensive necrotic core formation typically develops, thereby allowing assessment of disease-related phenotypes in a relatively stable culture environment 31. The immunofluorescence staining showed that all cortical neurospheres had many β3-tubulin-positive neurons (Figures 6B-E). PS1 F105C cortical neurospheres exhibited a significantly higher proportion of phosphorylated tau (AT8) compared to the isogenic WT control (Figures 6B-C). In addition, we used a specific antibody to detect Aβ42 and found that the expression of Aβ42 was also significantly elevated in F105C cortical neurospheres compared to its isogenic controls (Figures 6D-E). The knockout of APP from PS1 F105C cortical neurospheres also led to reduced expressions of AT8 and Aβ42 (Figures 6B-E), which were consistent with the results of 2D cortical neuron culture.

## Discussion

The parahippocampal gyrus, a cortical region critical for memory processing, undergoes early degeneration in AD and serves as a recognized biomarker strongly associated with clinical symptoms 32. In this study, transcriptomic and proteomic analyses of sporadic AD patient tissue revealed marked alterations in pathways linked to mitochondrial dysfunction within the parahippocampal gyrus. Notably, PS1 protein levels were significantly elevated in AD samples compared to controls, implicating PS1 in the development of mitochondrial abnormalities in sporadic AD.

Both PS1 and APP are central to AD pathogenesis, yet prior studies have largely emphasized their roles in γ-secretase-mediated cleavage and Aβ production, leaving their direct impact on mitochondrial function underexplored. To address this gap, we established human iPSC-derived cortical neurons carrying the PS1 F105C knock-in and A246E patient-derived mutations. These neurons not only reproduced hallmark AD phenotypes—such as tau phosphorylation and elevated Aβ42 and Aβ40 production—but also exhibited profound, intrinsic disruptions in mitochondrial function (Figure 6F). In addition, mitochondrial dysfunction was strongly associated with increased FL-APP expression and its aberrant mitochondrial localization, implicating APP as a potential mediator of PS1 mutant-induced mitochondrial impairment. Notably, APP KO in the mutant PS1 cells reduced mitochondrial function and attenuated AD-related features, including Aβ and tau accumulation. Collectively, these results highlight a previously underappreciated mechanism by which PS1 mutations drive neurodegeneration, bridging APP dysregulation with mitochondrial dysfunction in AD.

A well-known function of γ-secretase is the cleavage of the 99 amino acid C-terminal fragment (C99) of APP into Aβ, which subsequently is secreted and accumulates as plaques. Previous *in vitro* analysis of 138 FAD PS1 mutations indicated that most mutations resulted in the decreased Aβ generated from the γ-secretase cleavage of C99 fragment 33. Most studies were generated via overexpression of mutant PS1 in non-neuronal cell lines such as HEK293 cells and murine cell lines such as N2a, which may not have provided the accurate context for the generation of Aβ42 and Aβ40. In contrast, neurons derived from patients with FAD PS1 mutations and sporadic AD have shown to produce more Aβ 34-39. These contrasting results emphasized potential species and cell type specificity of Aβ production in AD and the importance of studying AD in a human-based model that closely mimics the cellular environment of the human brain. Human iPSC avoids species differences and more precisely recapitulates human AD development. Generating cortical neurons from iPSCs, which are the predominant cell type that undergoes neurodegeneration in AD, we observed a consistent increase in Aβ40 and Aβ42 across two PS1 mutations that are on different exonal regions, therefore suggesting the correct cell type may be required to observe bona fide Aβ40and Aβ42 generation.

Various AD phenotypes can be observed in human iPSC-derived neurons with FAD PS1 mutations as compared with healthy people iPSC-derived neurons. However, this approach introduces confounding factors in data interpretation due to the diversity of genetic background 40, 41. Therefore, the establishment of isogenic cells is necessary to deeply study AD phenotypes. CRISPR/Cas9 is a powerful genome-editing technology for generating isogenic iPSC models. It can be applied either to introduce PS1 mutations into the iPSC genome or to correct mutations in patient-derived iPSCs. In our previous work, we successfully used CRISPR/Cas9 to generate FAD PS1 F105C knock-in iPSCs from a healthy individual's line 42. In this study, we extend this approach by, for the first time, correcting the FAD PS1 A246E mutation in patient-derived iPSCs. This patient iPSC line is one of the most widely studied for FAD. Compared with uncorrected patient iPSC-derived neurons (A246E), the corrected isogenic neurons (Ctrl2) displayed markedly reduced AD-related phenotypes, including lower Aβ40 and Aβ42 production, decreased phosphorylated tau accumulation, and improved mitochondrial function. These findings demonstrate that pathogenic features of FAD patient cortical neurons carrying PS1 mutations can be effectively ameliorated through CRISPR/Cas9-mediated correction.

In our study, isogenic control iPSC-derived neurons maintained normal neuronal phenotypes, whereas cortical neurons and cortical neurospheres from PS1 mutant lines showed accumulation of hyperphosphorylated tau. Other FAD PS1 mutations have also been reported to lead to hyperphosphorylated tau accumulation in human neurons 43-46. This accumulation is thought to be associated to aberrant APP metabolism 47. Additionally, APP is involved in the endocytosis-mediated uptake of extracellular tau 48. Our results demonstrated that APP levels were significantly elevated in PS1 mutant neurons compared with isogenic control. Importantly, APP KO could reduce the accumulation of hyperphosphorylated tau in PS1 mutant neurons, supporting the hypothesis that APP plays a key role in tau pathology.

FAD PS1 F105C and A246E mutant iPSC-derived cortical neurons displayed early mitochondrial dysfunction. Recent study from MacMullen et al. also found similar mitochondrial dysfunction in FAD PS1 A246E, A97V, M139V, and E280A mutant iPSC-derived neurons 49. Previous studies have shown that APP can mislocalize to brain mitochondria, where its acidic domain (residues 220-290) interferes with protein translocation and traps APP within mitochondrial channels 50, 51. Specifically, APP has been shown to bind components of the TOM complex, including TOM20 and TOM40, thereby blocking the import of nuclear-encoded mitochondrial proteins 51. This import blockade leads to impaired respiratory chain assembly, reduced ATP production, elevated ROS, and loss of mitochondrial membrane potential—established hallmarks of mitochondrial dysfunction in AD 50. Extending these observations, we demonstrate that elevated FL-APP in mitochondria of PS1 mutant cortical neurons is associated with mitochondrial dysfunction. These results also align with those of Brooks et al., Minarjez et al., and Parker et al., who reported downregulation of OXPHOS proteins in postmortem sporadic AD hippocampal and frontal cortex samples, suggesting a potential interaction between the APP and mitochondrial function involved in the pathogenesis of FAD and sporadic AD. While the precise molecular mechanisms remain to be defined, such interactions could involve direct binding of N-terminal APP with mitochondrial proteins. Future studies should validate these findings across broader genetic backgrounds and explore whether modulating APP-mitochondria interactions can serve as a therapeutic strategy in early AD.

A key limitation of our study is that iPSC-derived neurons and neurospheres primarily reflect early developmental and maturation stages rather than late-onset neurodegeneration. While informative for uncovering early pathogenic mechanisms, these models do not fully capture the progressive nature of AD. Long-term cultures could help address this gap. However, they are technically challenging due to issues such as reduced viability, detachment in monolayer cultures, and necrotic core formation in neurospheres. Future studies employing improved long-term culture methods or more advanced 3D systems will be important to determine whether the effects of APP knockout persist throughout disease progression. Moreover, as we examined only two well-characterized PS1 mutations, future studies incorporating a broader spectrum of FAD-associated variants will be important for determining how generalizable these mitochondrial phenotypes are across FAD PS1 mutation-driven AD. Finally, as our APP knockout rescue experiments did not include a wild type line with APPKO, future studies will be needed to fully define the baseline effects of APP loss on mitochondrial function independent of PS1 mutations.

## Supplementary Material

Supplementary figures and tables 1-7.

Supplementary table 8.

Supplementary table 9.

## Abbreviations

AD: Alzheimer's disease
APP: amyloid precursor protein
Aβ: amyloid-β
iPSCs: induced pluripotent stem cells
PS1: presenilin-1
FAD: familial Alzheimer's disease
WT: wild-type
MSBB: Mount Sinai Brain Bank
TMM: M-values
CDR: clinical dementia rating scale
CERAD: Consortium to Establish a Registry for Alzheimer's Disease
DEG: differential gene expression
DEP: differential protein expression
BH: Benjamini-Hochberg
SEM: standard error of the mean
TMRE: tetramethylrhodamine, ethyl ester
OXPHOS: oxidative phosphorylation
FL-APP: full-length APP
KO: knockout
C99: 99 amino acid C-terminal fragment
