# Supplementary material for: APP Deficiency Ameliorates FAD Presenilin 1 F105C and A246E Mutations-induced Mitochondrial Dysfunction in Human Cortical Neurons
Source: Int J Biol Sci. 2026 Feb 18;22(5):2720–35. doi: 10.7150/ijbs.120062 (PMC12965328; doi:10.7150/ijbs.120062)
Supplement: Supplementary file 1 — Supplementary figures and tables 1-7. [file ijbsv22p2720s1.pdf]

Supplementary Figure Legends:

Supplementary Figure 1. Identified DEGs and DEPs in BM36 region from the MSBB study. (A)(B) Significant DEGs (FC > 1.2, Padj < 0.05) in BM36 according to four AD traits compared between each AD stages and Normal group. (C)(D) The overlapped 1,308 upregulated and 654 downregulated DEPs [abs(FC) > 1 and Padj < 0.05] which showed consistent alterations with the corresponding DEGs in BM36 region, according to four AD traits compared between each AD stages and Normal group.

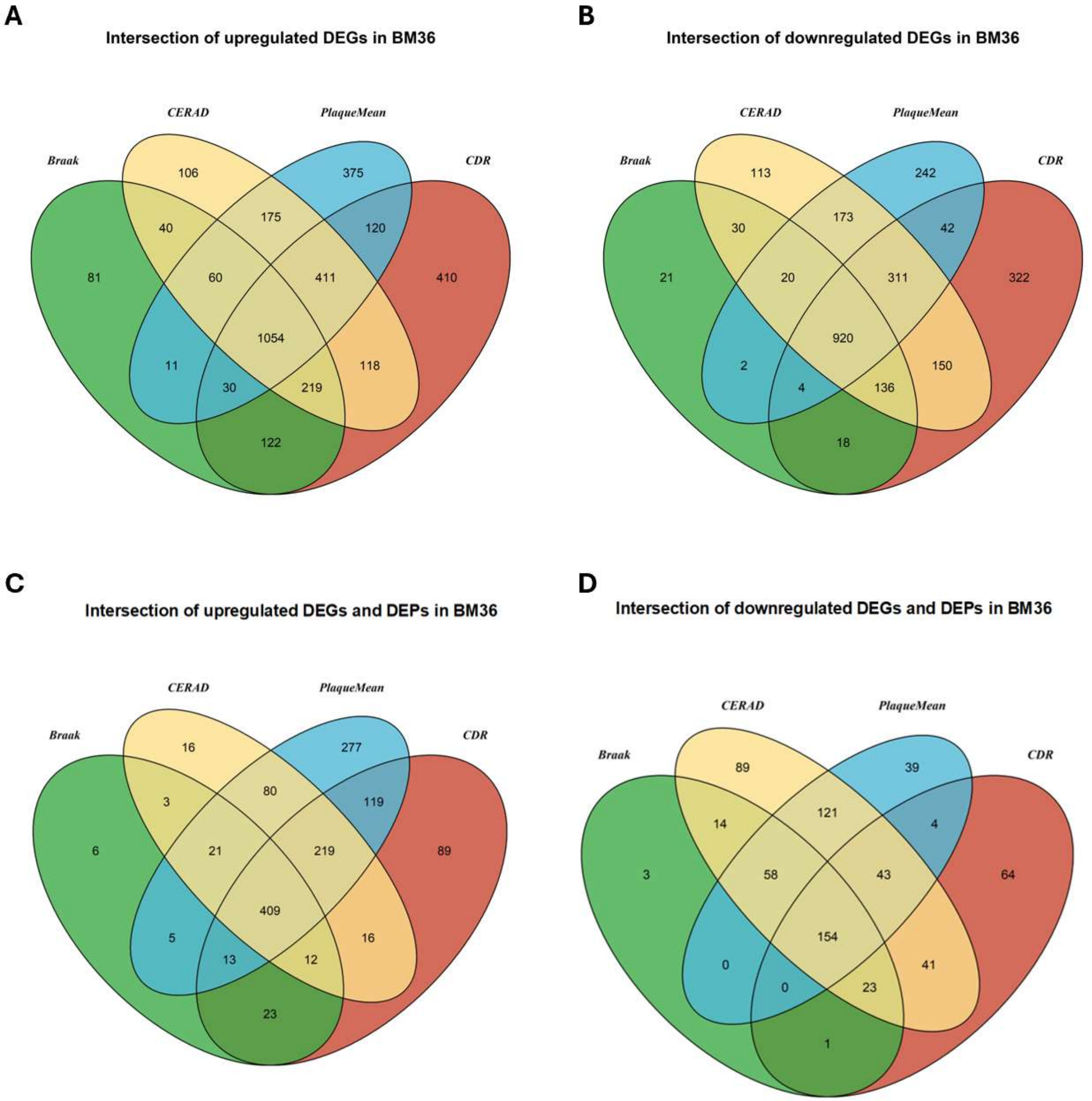

Supplementary Figure 2. The normalized mRNA levels of genes. (A) The normalized mRNA expression levels of PSEN1 across different groups according to four AD traits in four brain regions. (B-E) The normalized mRNA expression levels of NDUFB8, UQCRC2, SDHB, and MT-CO2 in BM36 across different groups according to four AD traits. comparisons of gene expression levels across multiple groups were conducted using one-way ANOVA followed by Dunnett's test for pairwise comparisons against the normal group. For comparisons between two groups, Student's t-test was employed. *P < 0.05, **P < 0.01, ***P < 0.001, ****P < 0.0001.

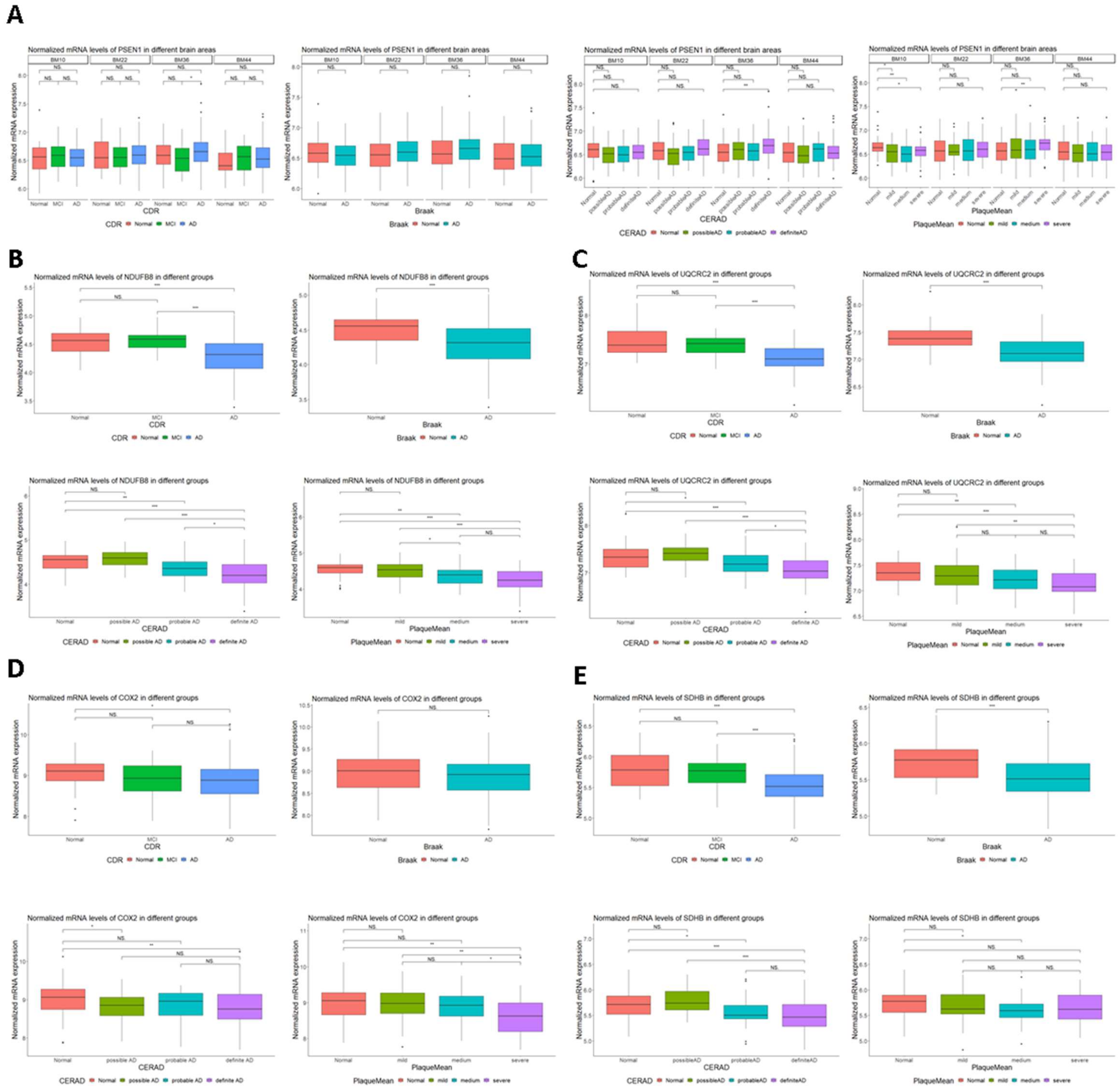

Supplementary Figure 3. Characterization of Ctrl 1, F105C, Ctrl 2, A246E, and APPKO iPSCs. (A) Schematic representation of the F105C and A246E mutations in PS1 protein. Asterisks represent the mutation sites in the transmembrane domain of PS1. Cross represents the endoproteolytic cleavage site of PS1. (B) Sanger sequencing was used to identify PS1 with F105C mutation and A246E mutation in iPSCs. (C) Sanger sequencing was used to identify APP KO site in PS1 F105C mutant iPSCs. (D) Immunofluorescence staining showed the expression of pluripotent stem cell-specific markers Oct4 and Nanog. (E) Normal karyotype of Ctrl 2 and APPKO iPSCs.

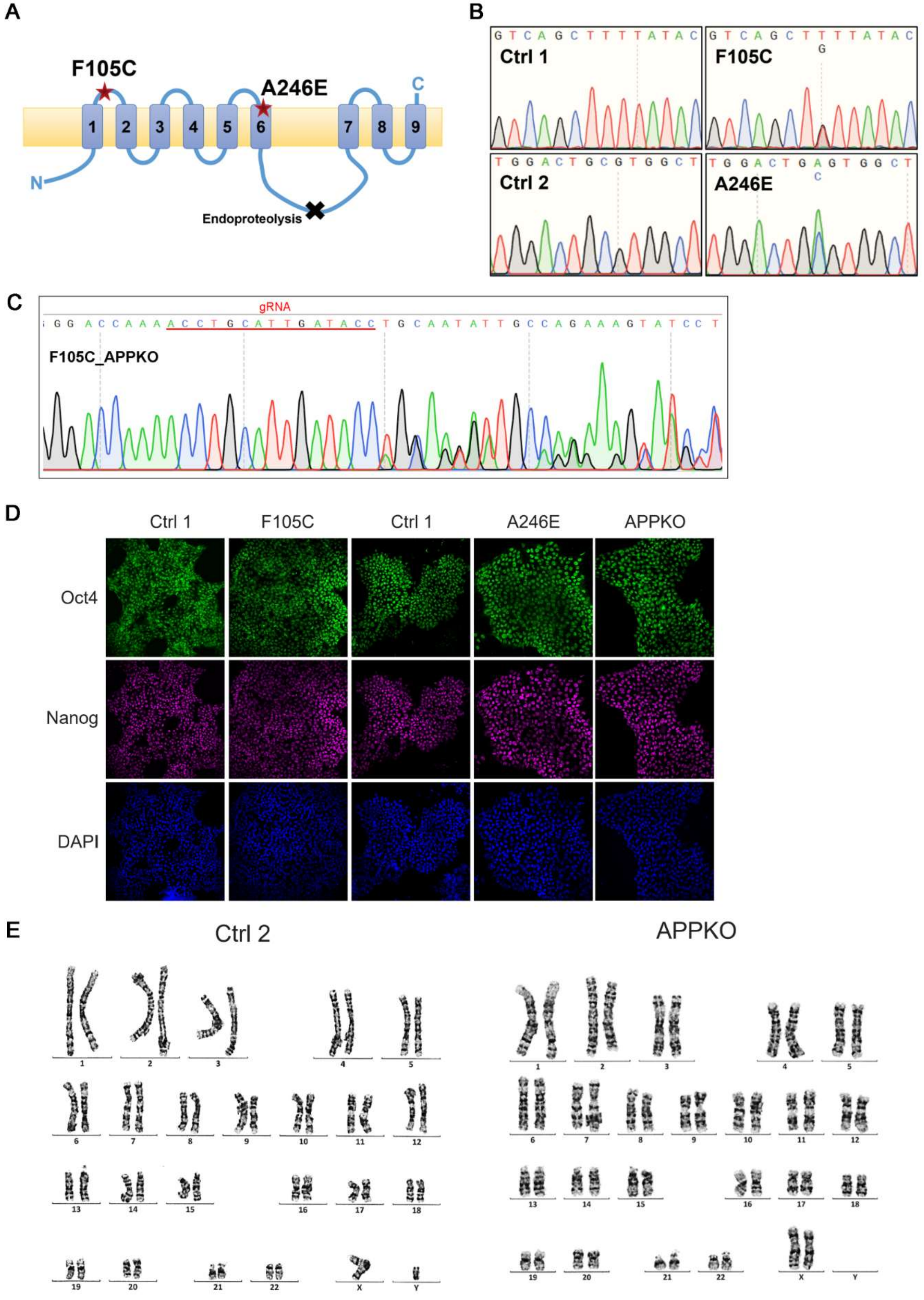

Supplementary Tables:

Supplementary Table 1.

Traits	Description	Group information
CDR	Clinical dementia rating	Normal: 0-2; AD: > 2
Braak	Braak neurofibrillary tangle score	Normal: 0; MCI: 0.5; AD: ≥ 1
CERAD	Neuropathology category	1) Normal: = 1; AD: > 1<br>2) Normal = 1, definite AD = 2, probable AD = 3, and possible AD = 4
PlaqueMean	The average of neuritic plaque density measures in five regions	Normal: 0; mild: > 0 & <= 6; medium: > 6 & <=12; severe: > 12

Supplementary Table 2. Primers for checking the mutation sites.

Primer	Sequence (5'-3')
F105C_F	GGAGCACAACGACAGACGG
F105C_R	AATGGCCCTGAGGTGGAAA
A246E_F	AGCACAGTTGATATAGGTTATGGTA
A246E_R	TGGGATGTACACGTTACCATTTT
APPKO_F	AAGGAGTGTTGAAGACCGGG
APPKO_R	ACGTGAATTGCTAGCCACCG

Supplementary Table 3: gRNA for the establishment of Ctrl 2 and F105C_APPKO iPSC lines.

gRNA	Sequence (5'-3')
PSEN1-E246A (Ctrl 2)	ATGGACTGAGTGGCTCATCT**TGG**
F105C_APPKO	ACCTGCATTGATACCAAGGA**AGG**

Red: NGG

Supplementary Table 4. ssDNA for the establishment of Ctrl 2.

ssDNA	Sequence (5'-3')
PSEN1-E246A (Ctrl2)	CCTGGTGTTTATCAAGTACCTCCCTGAATGGACT**GCG**TGGCTCATCTTGGCTGTGATTTCAGTATATGGTAAAACCCAAG

Green: Corrected site

Supplementary Table 5. Primers for off-target analysis

Name	Sequence
Ctrl 2_F1_OT1	TTCTTCCTCTGGGTTGATGG
Ctrl 2_R1_OT1	TACCTCATTGGCTTGGGAAG
Ctrl 2_F1_OT2	AATGTAGGCCCTGGTCCTCT
Ctrl 2_R1_OT2	TCTGGAGAGGATGGAGGAGA
Ctrl 2_F1_OT3	AATTGCAAGGCTGTCTGCTC
Ctrl 2_R1_OT3	GGTTCTGCTTCAGCTTTGCT
APPKO_F1_OT1	TTACAGGGGCCTGGGCAGAT
APPKO_R1_OT1	GGAGTGAATGCCTGCGTGAG
APPKO_F1_OT2	ACGGGCCAGGCATCCAAAGT
APPKO_R1_OT2	CAGAGCACTTGACCTTGGACG
APPKO_F1_OT3	CGGATAAGACTAGTTTGGGGCTC
APPKO_R1_OT3	AGCATTGTCAGGCCTGGTAAGC

Supplementary Table 6. Off-target analysis of Ctrl2 and F105C_APPKO iPSC lines. Three possible off-target sites predicted by COSMID were shown in the table. None, no off-target indels (insertion and deletion) were identified.

Lines	ID	Sequence	Mismatch	Location	Indels
Ctrl2	OT1	**G**TGGACTGA**T**TGGCTCATCTCAG	2	Chr1:76607989-76608008	none
Ctrl2	OT2	**T**TGGACTG**CCG**GGCTCATCTGAG	4	Chr8:144579729-144579748	none
Ctrl2	OT3	**G**TGG**T**CTG**C**GTGGCTC**T**TCTTAG	4	ChrY:8621212-8621231	none
F105C_ APPKO	OT1	**C**CCTGCAT**A**GATA**G**CAAGGAGGG	3	Chr12:51871903-51871925	none
F105C_ APPKO	OT2	**T**CCTGCAT**G**GATA**A**CAAGGACGG	3	ChrX:154359326-154359348	none
F105C_ APPKO	OT3	A**T**CTGCA**G**TGA**A**ACCAAGGAGGG	3	Chr2:236332916-236332938	none

Red: Mismatch

Supplementary Table 7: Antibodies used for immunofluorescence (IF) and western blot (WB).

Antibody	Company & Product code	Application
MAP2	Sigma-Aldrich, M1406-02ML	IF 1:400
Neurofilament	Abcam, ab7794	IF 1:500
NeuN	Invitrogen, 702022	IF 1:500
TBR1	Abcam, ab31940	IF 1:200
AT8	Invitrogen, MN1020	WB 1:1000, IF 1::1000
Tau	CST, 4019	WB 1:1000
GAPDH	Invitrogen, MA5-15738	WB 1:1000
TOM20	Abcam, ab78547	IF 1:1000
DRP1	CST, 5391	WB 1:1000
FIS1	Invitrogen, PA5-22142	WB 1:1000
OPA1	CST, 80471	WB 1:1000
MFN1	CST, 14739	WB 1:1000
OxPhos Human WB Antibody Cocktail	Invitrogen, 45-8199	WB 1:1000
APP (22C11)	eBioscience™, 14-9749	WB 1:1000, IF 1:250
APP (NAB228)	CST, 2450	IF 1:250
Myc-tag	CST, 2272	WB 1:1000, IP 1:400
beta Amyloid (1-42)	Invitrogen, 44-344	IF 1:200
βIII-tubulin	Sigma-Aldrich, T8660	IF 1:1000
Nanog	R&D, AF1997	IF 1:200
Oct4	Proteintech, 11263-1-AP	IF 1:500
